# Development of a SNP linkage map and genome-wide association study for resistance to *Aeromonas hydrophila* in pacu (*Piaractus mesopotamicus*)

**DOI:** 10.1186/s12864-020-07090-z

**Published:** 2020-09-29

**Authors:** Vito A. Mastrochirico-Filho, Carolina H. S. Borges, Milena V. Freitas, Raquel B. Ariede, Fabiana Pilarski, Ricardo Utsunomia, Roberto Carvalheiro, Alejandro P. Gutierrez, Carolina Peñaloza, José M. Yáñez, Ross D. Houston, Diogo T. Hashimoto

**Affiliations:** 1 São Paulo State University (Unesp), Aquaculture Center of Unesp, Via de Acesso Prof. Paulo Donato Castellane, s/n, Jaboticabal, SP 14884-900 Brazil; 2 Universidade Federal Rural do Rio de Janeiro, Seropédica, RJ 23897-000 Brazil; 3 São Paulo State University (Unesp), School of Agricultural and Veterinarian Sciences, Jaboticabal, SP Brazil; 4 National Council for Scientific and Technological Development (CNPq), Brasília, DF 71605-001 Brazil; 5 The Roslin Institute and Royal (Dick) School of Veterinary Studies, The University of Edinburgh, Midlothian, EH25 9RG UK; 6 Facultad de Ciencias Veterinarias y Pecuarias, Universidad de Chile, Santiago, Chile

**Keywords:** Motile Aeromonas septicemia, Disease outbreaks, Resistance QTLs, Fish farming

## Abstract

**Background:**

Pacu (*Piaractus mesopotamicus*) is one of the most important Neotropical aquaculture species from South America. Disease outbreaks caused by *Aeromonas hydrophila* infection have been considered significant contributors to the declining levels of pacu production. The current implementation of genomic selection for disease resistance has been adopted as a powerful strategy for improvement in fish species. This study aimed to investigate the genetic architecture of resistance to *A. hydrophila* in pacu via Genome-Wide Association Study (GWAS), the identification of suggestive Quantitative Trait Loci (QTLs) and putative genes associated with this trait. The genetic data were obtained from 381 juvenile individuals belonging to 14 full-sibling families. An experimental challenge was performed to gain access to the levels of genetic variation for resistance against the bacteria using the following trait definitions: binary test survival (TS) and time of death (TD).

**Results:**

The analyses of genetic parameters estimated moderate heritability (*h*^*2*^) for both resistance traits: 0.20 (± 0.09) for TS and 0.35 (± 0.15) for TD. A linkage map for pacu was developed to enable the GWAS, resulting in 27 linkage groups (LGs) with 17,453 mapped Single Nucleotide Polymorphisms (SNPs). The length of the LGs varied from 79.95 (LG14) to 137.01 (LG1) cM, with a total map length of 2755.60 cM. GWAS identified 22 putative QTLs associated to *A. hydrophila* resistance. They were distributed into 17 LGs, and were considered suggestive genomic regions explaining > 1% of the additive genetic variance (AGV) for the trait. Several candidate genes related to immune response were located close to the suggestive QTLs, such as *tbk1*, *trim16*, *Il12rb2* and *lyz2*.

**Conclusion:**

This study describes the development of the first medium density linkage map for pacu, which will be used as a framework to study relevant traits to the production of this species. In addition, the resistance to *A. hydrophila* was found to be moderately heritable but with a polygenic architecture suggesting that genomic selection, instead of marker assisted selection, might be useful for efficiently improving resistance to one of the most problematic diseases that affects the South American aquaculture.

## Background

Bacterial diseases are responsible for the loss of billions of dollars to aquaculture production every year, and may be considered a major threat to the fish farm’s sustainability [1, 2]. Motile *Aeromonas* septicemia is a common infectious disease in aquaculture caused by *Aeromonas hydrophila*, a gram-negative bacterial pathogen, which is generally associated with symptoms such as reddened or rotten fins, external/internal septicemia, and hemorrhage of aquatic organisms [3].

The incidence of sanitary problems associated to the intensification of fish production have caused large mortalities by *A. hydrophila* infection that involves important farmed fish species from South America, such as pacu (*Piaractus mesopotamicus*) [4, 5]. Although there are no official statistics about the economic losses related to mass mortalities caused by *A. hydrophila* outbreaks at the pacu farms, non-official communications of Brazilian farmers have reported that *A. hydrophila* infection is responsible for total mortalities ranging between 20 to 30% of the annual production [6].

While practically all Neotropical fish production is still carried out based on unselected stocks, large-scale genetic studies performed on global aquaculture species, such as carp [7–9], tilapia [10] and salmonids [11–13] have highlighted major possibilities to improve resistance to infectious diseases by selective breeding. In contrast, selective breeding for disease resistance is challenging because this trait is difficult to measure directly on selection candidates [14, 15]. While major QTLs affecting disease resistance have been identified in some studies [16, 17], such traits typically exhibit a polygenic architecture. As such, it is preferable to have a dense genetic marker panel containing thousands of SNPs to combine linkage analysis and GWAS, in order to detect QTLs and identify candidate genes that are linked to the disease resistance trait [18, 19]. Current advances in understanding the genetic architecture of resistance against *A. hydrophila* by GWAS-based conclusions have already been performed in some fish species, including carp *Labeo rohita* [20] and catfish hybrids between *Ictalurus* species [21]. In both studies, several significantly associated QTLs to resistance against *A. hydrophila* were identified, containing candidate genes related to the immune responses.

Experimental challenges that consisted of an infection induced on pacu families have shown significant genetic variation for resistance against *A. hydrophila* when binary survival status and time of death were used as trait definitions, which have demonstrated the potential of the genetic improvement for *Aeromonas* resistance in this species [6]. However, GWAS analyses to understand the genomic basis of resistance to *A. hydrophila* have not yet been performed in pacu. Moreover, there is neither a dense SNP platform nor a genetic map available to perform GWAS analyses in pacu. Based on this proposal, restriction site-associated DNA sequencing (RAD-Seq) represents an affordable and viable technique for the discovery and genotyping of several sets of SNPs, through a reduced representation of the genome belonging to multiplexed libraries of samples [22–24], particularly for non-model species without reference genome, such as pacu.

The present study aimed for the discovery and the genotyping of SNPs using RAD-Seq approach on 14 full-sib pacu families, which had been experimentally challenged with *A. hydrophila* infection. These data were used to investigate the genetic architecture of resistance to this disease generating a medium density linkage map and GWAS analyses for host resistance, following survival and time of death as trait definitions. The presented results will be fundamental to evaluate the potential of genome-wide molecular information for obtaining subsidies on application of genetic selection studies aiming at resistance against *A. hydrophila*, and accelerating the genetic improvement of this trait on pacu aquaculture.

## Results

### Genetic parameter analysis

**Fig. 1:**
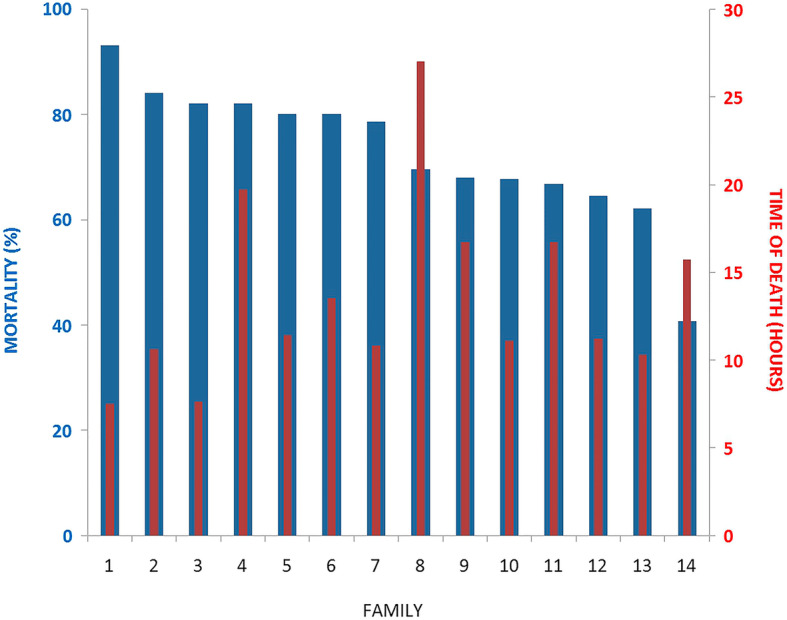
Mortality rate (%) and time of death (hours) of 14 pacu (*P. mesopotamicus*) families infected with *A. hydrophila* during 14-day resistance challenge. Blue columns represent mortality rates (%) for each family, whereas red columns represent the average time of death (hours) corresponding for each family

Before the challenge, all fish were healthy without any symptom of infection. Nevertheless, the presence of bacterial infection was checked in a random subsample by routine microbiological investigation. During the challenge test, susceptible individuals demonstrated lethargic behavior, erratic swimming and red spotted skin lesions over the operculum, head, fins and gills. No symptoms associated with infection were reported in individuals belonging to the unchallenged control group. High mortality rates were observed mainly at the second day after inoculation (Fig. 1). Considering the 381 challenged individuals, the total cumulative mortality rate at the end of the test period was 72.3% (277 individuals) across all the families. The cumulative mortality rate of the most susceptible family to *A. hydrophila* infection was 93.1% (family 1); in contrast, the most resistant family presented 40.7% of cumulative mortality rate (family 14), which indicated a considerable phenotypic variation associated to resistance against *A. hydrophila* infection (Fig. 1).

Additive-genetic variation was observed in both traits. Variance components and estimated heritabilities for TS and TD are presented in Table 1. The results registered moderate heritability values for *A. hydrophila* resistance in this experimental population of pacu, which were estimated in 0.20 (± 0.09) and 0.35 (± 0.15) for TS and TD, respectively. Heritability using the genomic information was similar to the pedigree-based results (data not shown).

**Table 1:** Estimates of additive genetic variance $\left({\sigma}_a^2\right)$, residual variance $\left({\sigma}_e^2\right)$, phenotypic variance $\left({\sigma}_p^2\right)$ and heritability (*h*^2^) for resistance to *A. hydrophila* in pacu (*P. mesopotamicus*), measured as test survival (TS) and time of death (TD). Standard error in parenthesis

Variance components	TS	TD
${\sigma}_a^2$	0.25 (0.14)	0.82 × 10^6^ (0.42 × 10^6^)
${\sigma}_e^2$	1	0.15 × 10^7^ (0.26 × 10^6^)
${\sigma}_p^2$	1.25 (0.14)	0.23 × 10^7^ (0.25 × 10^6^)
*h*^2^	0.20 (0.09)	0.35 (0.15)

### Identification of SNPs from RAD-Seq technology

Initially, 1,599,222,848 RAD-Seq reads with 150 bp were obtained. From the total, 795,613 low quality reads were excluded of the analysis. Additionally, 984,501,898 ambiguous barcodes and 11,344,436 ambiguous RAD tags were discarded, which resulted in 602,580,901 retained reads.

The average number of putative RAD loci identified for each family ranged from 37,035.12 (SD 4657.77) to 31,382.57 (SD 6664.41), with an average coverage ranging from 19x (SD 8x) to 25x (SD 15x). A catalog containing 246,566 consensus loci was created, of which 26,597 polymorphic loci with 162,305 SNPs were obtained. Only 30,093 SNPs presenting MAF values higher than 0.05, and on Hardy-Weinberg equilibrium were retained. SNPs that exceeded 20% of missing genotype data were also removed, leaving 18,262 SNPs for downstream analyses. Furthermore, 46 individuals exceeded 30% of missing genotype data and were discarded, which resulted in 345 individuals for a correct assignment of true parents.

### Parentage assignment

The pedigree information provided by the SNP data was tested for each individual to confirm a correct parental assignment. Considering the genotype errors found, it was not possible to obtain a conclusive analysis in 13 individuals due to the lack of statistical criteria (low likelihood ratio) and, therefore, were disregarded. Additionally, Mendelian rate test was carried out in the offspring to discard alleles that could not have been received them from their putative biological parents. Then, this analysis discarded 300 SNPs with more than 10% of Mendelian error rate, remaining 17,962 SNPs to be included in the linkage map.

### Linkage map and synteny analysis

As pacu has 27 pairs of chromosomes (2n = 54), 17,453 SNPs were assigned to 27 linkage groups. Initially, 652 SNPs presented none association to any linkage group. Posteriorly, 143 markers were joined to already existing linkage groups, and 509 markers were discarded because no association to the linkage map was detected.

**Fig. 2:**
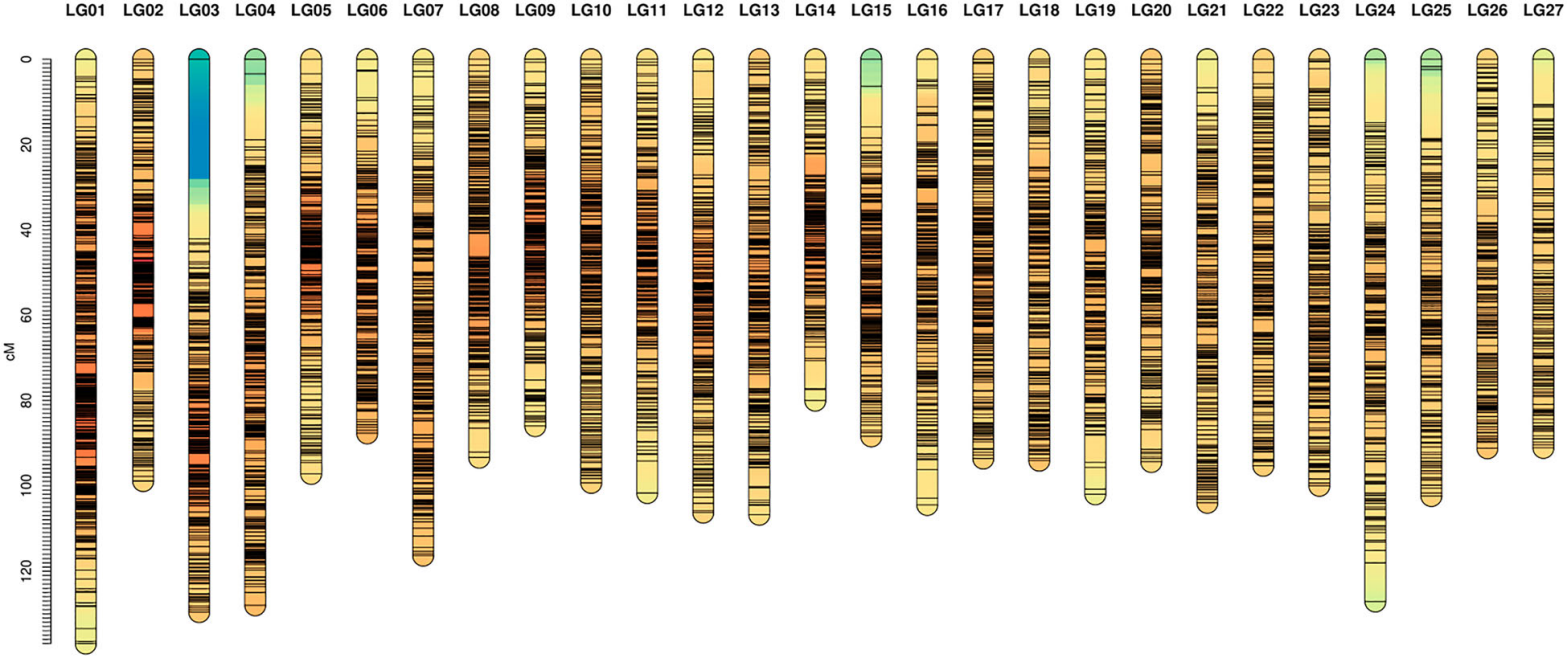
Linkage map of challenged families of pacu against *A. hydrophila* infection showing the 27 linkage groups and position of 17,453 SNPs. Colours represent a density scale of markers ranging from blue (low density regions) to red (high density regions)

To produce the linkage map, the *OrderMarkers2* module was repeated 5 times retaining the ordering with the highest likelihood for each linkage group. The marker orders with the highest likelihoods for each linkage group were combined to produce the final linkage map. In the ordering of markers, the number of SNPs varied from 262 (LG27) to 1504 (LG1) (Fig. 2).

The length of linkage groups varied from 79.95 (LG14) to 137.01 (LG1) cM, with a total integrated map length of 2755.60 cM and an average distance between markers of 0.47 cM. Considering the density within linkage groups, the linkage group that presented the highest and lowest density of markers were LG8 and LG24, with an average distance of markers of 0.38 and 0.66 cM, respectively (Fig. 2).

**Fig. 3:**
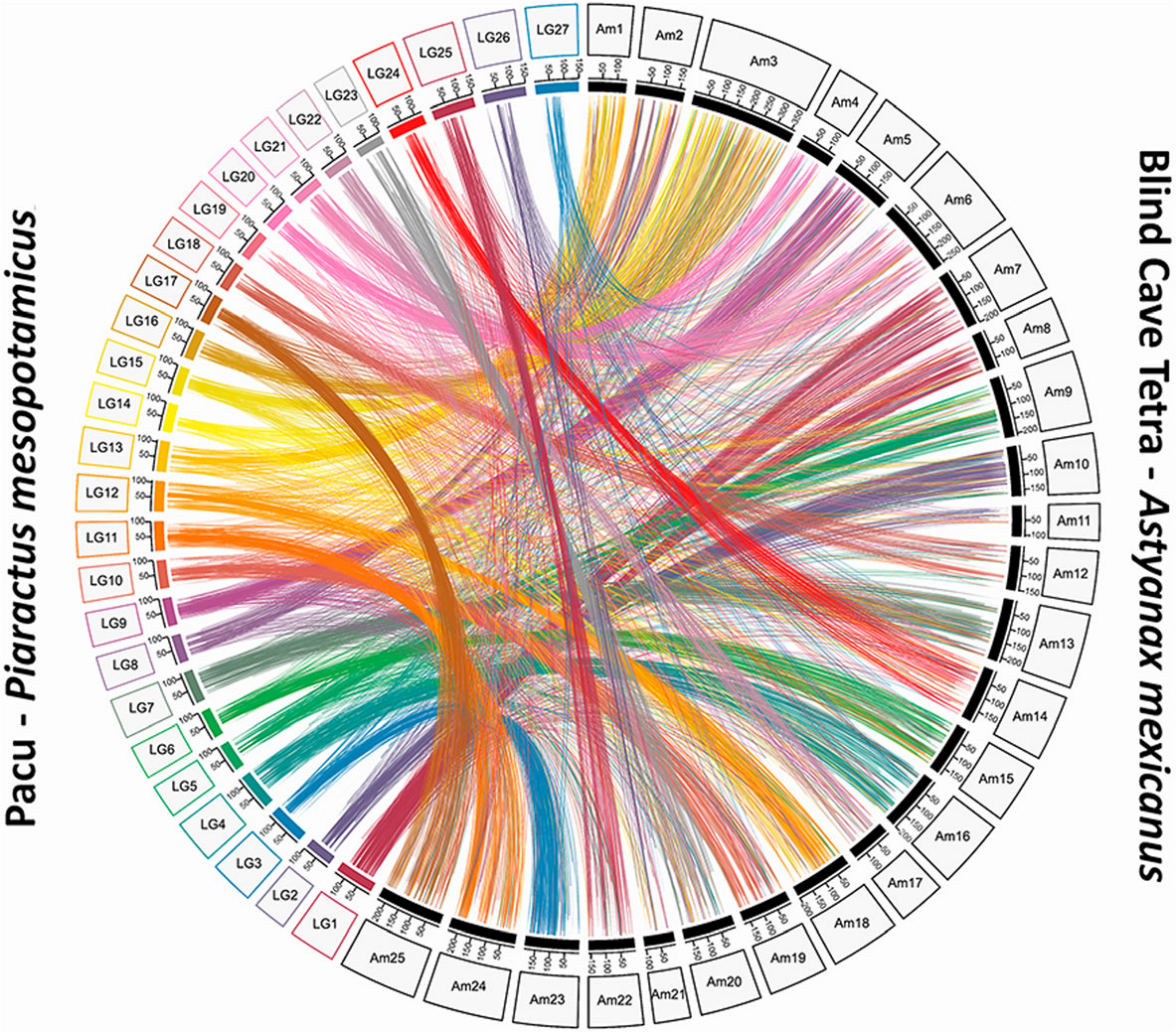
Genomic synteny showed by Circos diagram for each pair of alignments between the linkage groups of pacu (*Piaractus mesopotamicus*) (LG) and the chromosomes of blind cave tetra species (*Astyanax mexicanus*) (Am)

Conserved genomic synteny between pacu and the blind cave tetra (*Astyanax mexicanus*) species was investigated using all mapped loci on the LGs (10,220 loci). Significant BLASTn hits to blind cave tetra genome matched 2851 pacu RAD tags (27.9%) wherein 2194 (21.5%) showed synteny for all 27 linkage groups (Fig. 3). Despite the small percentage of alignments to the blind cave fish genome, a high level of genomic synteny was observed between pacu and this model species. The number of aligned segments for each linkage group ranged from 187 (LG1) to 22 (LG19). A 1:1 relationship was detected on at least 21 LGs (77.7%), excluding six LGs (LG1, LG8, LG14, LG19, LG26 and LG27) that obtained few percentages of mapped markers in a unique chromosome (equal to or less than 50% of the pacu segments). LG11 obtained the largest synteny relationship (83% of SNPs) to a unique chromosome (Am24), followed by LG23 to the chromosome Am20 (82.5% of SNPs). However, LG1 showed synteny to two chromosomes (Am7 and Am8), whereas three LGs (LG14, LG15 and LG16) were merged to a unique chromosome (Am3). Additionally, genomic synteny was not conclusive for two LGs (LG19 and LG27), showing a poor synteny relationship with any chromosome of the blind cave tetra species (Fig. 3).

### Genome wide association analysis (GWAS)

**Fig. 4:**
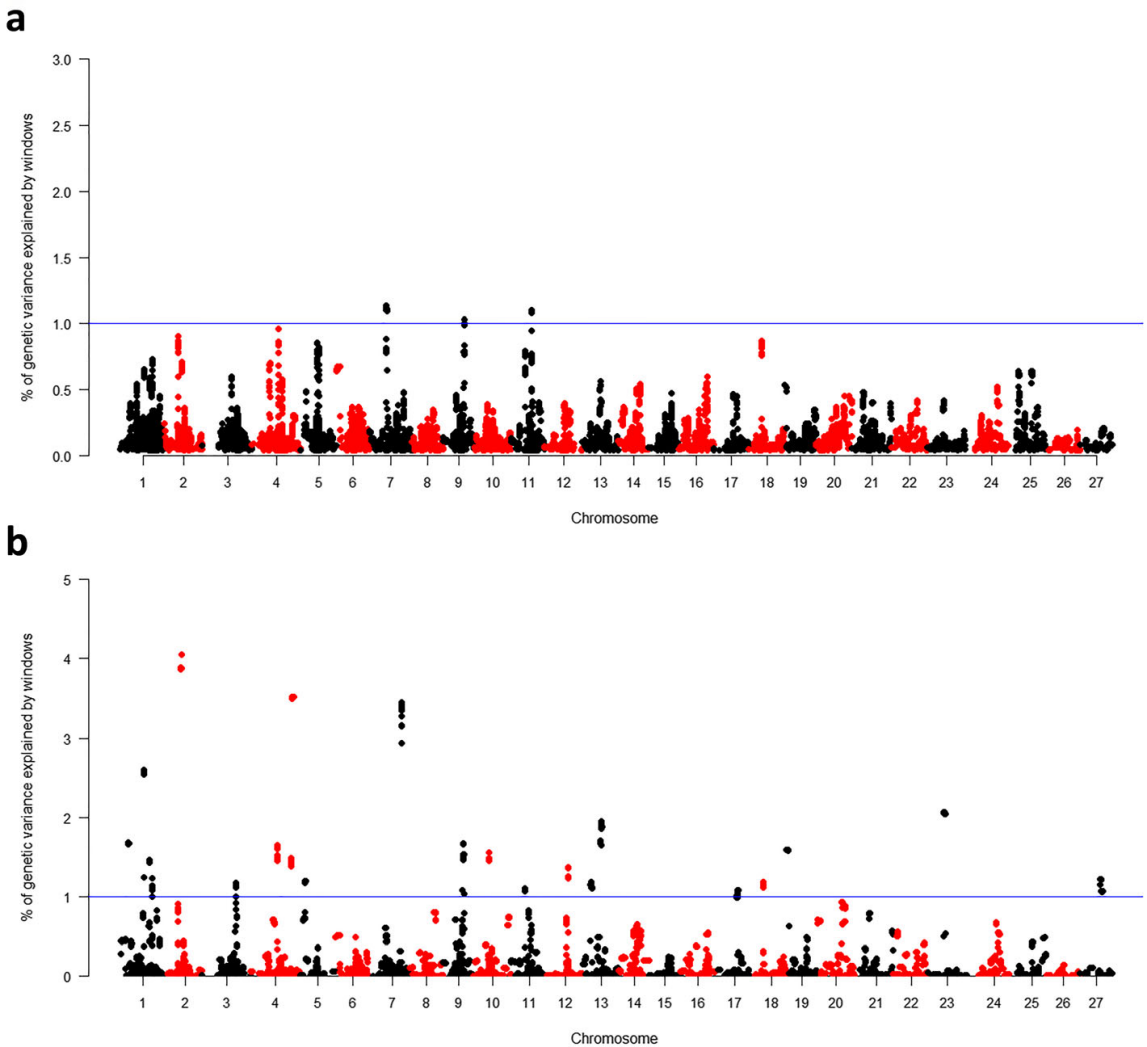
Genomic association analysis (GWAS) for test survival (TS) against *A. hydrophila* infection in 14 pacu families by GBLUP (**a**) and wGBLUP (**b**) models

**Fig. 5:**
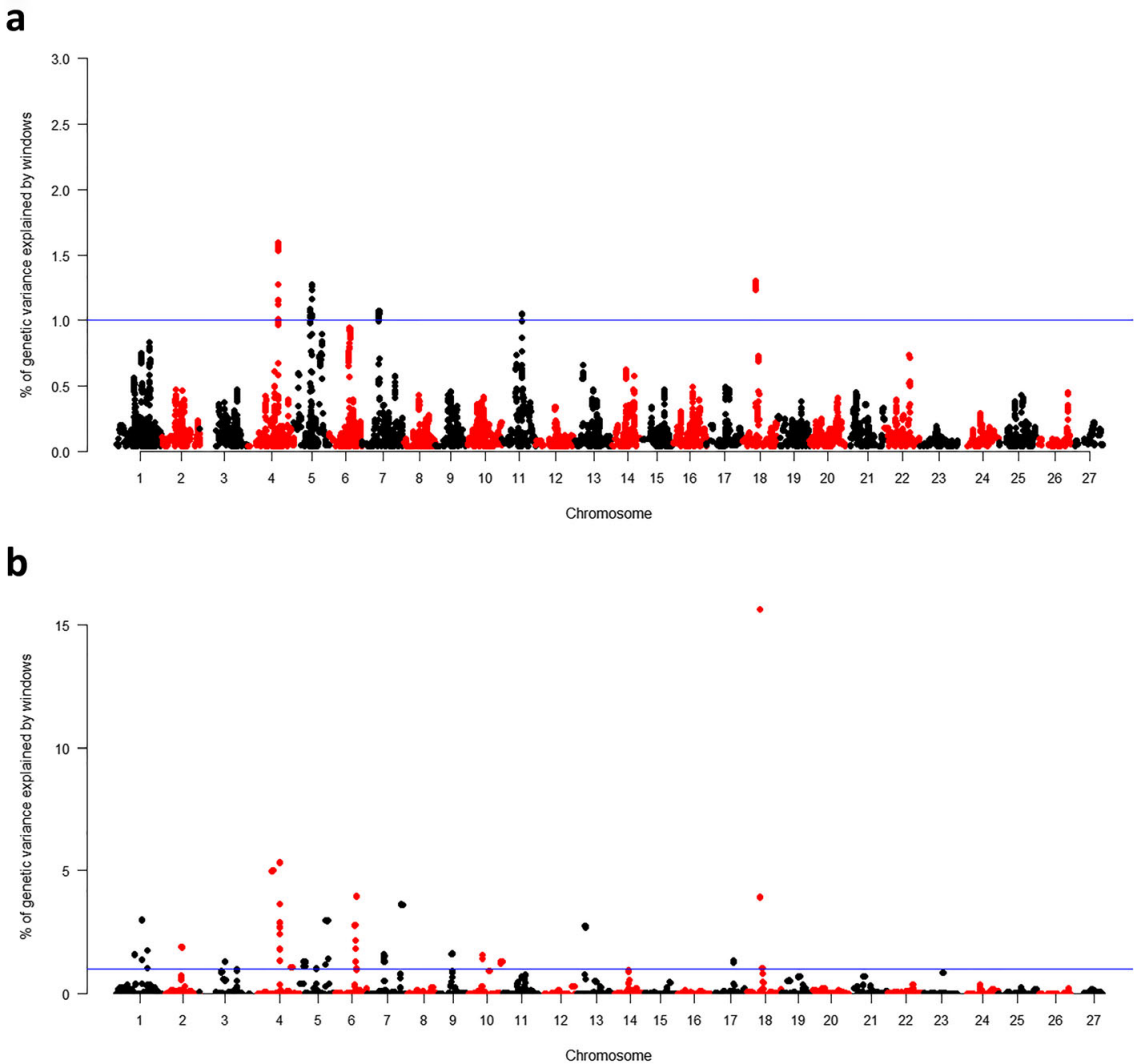
Genomic association analysis (GWAS) for time of death (TD) against *A. hydrophila* infection in 14 pacu families by GBLUP (**a**) and wGBLUP (**b**) models

In total, 22 suggestive QTLs presenting association (AGV > 1%) with *A. hydrophila* resistance were detected in 17 linkage groups, of which 5 were only associated with TS (LGs 12, 13b, 19, 23 and 27), 3 only associated with TD (5b, 6 and 10b), and 14 were registered in association for both traits (1, 2, 3, 4a, 4b, 5a, 7a, 7b, 9, 10a, 11, 13a, 17 and 18) (Table 2, Figs. 4 and 5). Additionally, 38 immune system genes were located close to the identified QTLs (Table 2).

**Table 2:** SNP markers associated with test survival (TS) and time of death (TD) traits estimated on pacu against *Aeromonas hydrophila* infection, using GBLUP and wGBLUP methods

QTL	GWAS method	AGV (%)		Genes related to the immune system
		TS	TD	
1	wGBLUP	2.59	2.99	*anp32b, srebf2*
2	wGBLUP	4.05	1.89	*acbd5, rala, zkscan1*
3	wGBLUP	1.17	1.31	*lrrc3b, myo16*
4a	wGBLUP	3.52	1.32	*camlg, tcf7, nlrc3*
4b	GBLUP	–	1.59	*tnfsf13b*
	wGBLUP	1.65	5.34	
5a	wGBLUP	1.19	1.40	*axl, emilin1*
5b	GBLUP	–	1.28	*kalrn, il4i1*
	wGBLUP	–	2.98	
6	wGBLUP	–	3.96	*lgr6*
7a	GBLUP	1.13	1.07	*cyp27c1, fundc1*
	wGBLUP	–	1.58	
7b	wGBLUP	3.45	3.63	*inhbb, srcin1, tcaf1*
9	GBLUP	1.03	–	*tbk1, trim16, zmat3*
	wGBLUP	1.67	1.62	
10a	wGBLUP	1.56	1.57	*mtmr10*
10b	wGBLUP	–	1.42	*–*
11	GBLUP	1.10	1.05	*rps6ka5, ccr3, cyp2k1*
	wGBLUP	1.19	–	
12	wGBLUP	1.14	–	*–*
13a	wGBLUP	1.18	2.74	*itpk1, mark4*
13b	wGBLUP	1.95	–	*myo18a, caln1*
17	wGBLUP	1.08	1.34	*tiam1*
18	GBLUP	–	1.30	*lyz2, il12rb2*
	wGBLUP	1.16	15.65	
19	wGBLUP	1.58	–	*unc5b, znf3*
23	wGBLUP	2.06	–	*slk*
27	wGBLUP	1.22	–	–

## Discussion

Studies on selection of superior genotypes related to resistance have been reported in several aquaculture species [13, 25, 26], however, no prior studies have focused on species produced in the Neotropical region. The major contribution of this study was to generate and apply genomic tools to improve the understanding of the genomic architecture of resistance against *A. hydrophila* in pacu, as a key step to harness genomic information to improve selection processes directed to disease resistance in this Neotropical fish species. Recently, dense SNP arrays have facilitated researchers assessing the association between genome-wide markers and utilizing them for optimization of the disease resistance through genetic improvement in aquaculture [21, 27–29]. However, whereas considerable investment for the development and application of dense SNP arrays are required, techniques such as RAD-Seq require fewer resources, especially when directed for smaller scale programs, offering a viable alternative to identify and apply SNP datasets for breeding program studies [30]. Recent studies have demonstrated that RAD-Seq allowed the identification of some QTLs associated with bacterial cold-water disease in rainbow trout [31], as well as to resistance to viral nervous necrosis in European sea bass [24].

### Genetic parameters

The results of genetic parameters revealed significant genetic variation for *A. hydrophila* resistance, with moderate heritability values (both to pedigree and genomic-based data). The heritability estimates were higher than those previously performed in different pacu populations (*h*^2^ = 0.15 for TS and *h*^2^ = 0.12 for TD) [6]. In general, our results were within the reference range of heritability previously reported for *A. hydrophila* resistance in other species, such as *Clarias macrocephalus*, *Megalobrama amblycephala*, and *Labeo rohita*, ranging from 0.12 to 0.39 [32–34]. Nevertheless, lower values have also been found for *A. hydrophila* resistance in rohu carp (*Labeo rohita*) and common carp (*Cyprinus carpio*), with values of 0.02 and 0.04, respectively [32, 35]. Therefore, the heritability values achieved for both resistance traits suggest that they will respond satisfactorily to genetic selection.

### Linkage map and synteny analysis

In this study, we report genome-scale SNP discovery and medium-density genetic map construction in pacu. These genomic tools enable analysis of productive traits (e.g., disease resistance) by performing GWAS analysis and providing insights on the genetic architecture of these production traits, as proposed in this study approaching the resistance to *A. hydrophila* in pacu. The linkage map was comprised of 27 linkage groups that correspond to the number of chromosomes of pacu, arranging 10,220 loci (Fig. 2). In terms of Neotropical fish species, the only available linkage map was constructed for tambaqui *Colossoma macropomum* [36], a species from the same group (Serrasalmidae family). Our results were structurally similar to the linkage map constructed for tambaqui (*C. macropomum*), which used the information of 7734 SNPs to obtain a map length of 2811 cM, with an average marker interval of 0.39 cM. Here, we prefer to use the genome of the blind cave tetra (*A. mexicanus*) as the reference genome to perform the synteny analysis instead of zebrafish, because the former is more related to pacu than the latter, which resulted in considerable homology between the LGs of pacu and chromosomes of *A. mexicanus*. A conserved synteny on the majority of LGs was identified between pacu and *A. mexicanus* genomes (Fig. 3), and a weak syntenic relationship was found in only 6 LGs. Therefore, although the reference genome of pacu is still not available, the resulting linkage map with conserved synteny to the genome of *A. mexicanus* will offer a framework for mapping candidate genes responsible for productive traits to be included in breeding programs of pacu.

### Genome wide association analysis (GWAS)

In total, 22 suggestive QTLs associated to resistance against *A. hydrophila* (AGV > 1%) were observed in 17 linkage groups (Figs. 4 and 5). This pattern of polygenic architecture was similarly found in other fish species challenged for *A. hydrophila* resistance [20] and for other bacterial diseases [25, 37].

Marker-assisted selection has been suggested as a viable approach for catfish breeding due to the limited number of QTLs involved in *A. hydrophila* resistance (3 QTLs presented in 3 linkage groups) [21]. However, the absence of large effect QTLs suggests that marker-assisted selection cannot be considered an effective strategy to genetically improve resistance against *A. hydrophila* in the analyzed population of pacu, similarly to a previous QTL mapping study that associated 21 QTLs to 10 linkage groups for *A. hydrophila* resistance in rohu carp [20]. Therefore, the application of genomic selection [38] could be tested for selection accuracy of breeding values compared to traditional pedigree-based selection, and it can be used as an alternative to increase the selection response for disease resistance in pacu, as similarly performed for resistance of salmonid species [11, 13, 19, 28, 39].

Among the QTLs with highest AGV, six were identified adopting both models GBLUP and wGBLUP (4b, 5b, 7a, 9, 11 and 18) (Figs. 4 and 5). These six suggestive QTLs were located close to important candidate genes related to immune response, which may probably be involved in resistance against *A. hydrophila* infection (Table 2). Some of these genes were already reported in several studies involving the functioning of immune system mechanisms of fish in face of bacterial infections [40], corroborating the results of the present study and suggesting similar mechanisms for bacterial resistance in different fish species. As example, *TANK-binding kinase 1-binding protein 1* (*tbk1*) gene had an essential role in the activation of pattern-recognition receptors, starting the innate immune responses against pathogens [41]. *Tripartite motif-containing protein 16* (*trim16*) gene is a part of tripartite motif family which has also been associated with QTL regions for *A. hydrophila* resistance in the hybrid catfish [21]. Several studies have showed the involvement of this family of genes with signaling pathways for activation of murine macrophages [21], autophagic response [42], and interleukin secretion [43]. *Interleukin 12 Receptor Subunit Beta2* (*Il12rb2*) is responsible in regulation of receptors to interleukin-12, a proinflammatory cytokine produced by phagocytic cells already identified in response to *A. hydrophila* infection [44]. Additionally, this gene is involved in the proliferation of T-cells as well as NK cells, enhancing the cytotoxic activity against the bacteria [45]. *Lysozyme C-2 precursor* (*lyz2*) is another gene responsible to contribute to the inflammatory response*,* as it is associated to activity of immune modulating agents and digestive function against gram-negative bacteria [46]. This gene has already been found in several fish species, such as salmonids, japanese flounder and upregulated in grass carp challenged with *A. hydrophila* [40, 47]. Therefore, further studies are needed on validation of these predicted genes as important components in the role of the genetic variation for resistance against *A. hydrophila*.

## Conclusion

There have been several studies aiming to improve disease resistance via analysis of high-density SNP genotypes in aquaculture [13, 25, 26], but until the present date, no studies have focused on species produced in the Neotropical region. The present study generated thousands of SNPs for pacu, which were used to identify the genetic architecture of *A. hydrophila* resistance through GWAS analyses, highlighting the effectiveness of genotyping-by-sequencing techniques for genomic analysis in non-model fish species. Our results suggest that this trait is under polygenic control in pacu. Thus, genomic selection might be suitable to incorporate molecular information to aim for the improvement of resistance to one of the most problematic diseases that affects the South American aquaculture.

## Methods

### Origin of pacu families

Genomic data were obtained from 381 juvenile individuals (6 months post hatch) belonging to 14 full-sibling families of pacu. The induced reproduction was generated in 2016 by a hierarchical mating scheme using 4 dams and 14 sires (approximately 1 dam for each 4 sires). These breeders were originated from three different fish farms located at Sao Paulo State (Brazil). The names of the fish farms were kept confidential. Induced spawning was successfully carried out injecting carp pituitary extract, similarly to Mastrochirico-Filho et al. [6].

After incubation of fish embryos and hatching process, artemia were offered to the larvae during 20 days in 20 l conical fiberglass incubators installed in the Laboratory of Genetics in Aquaculture and Conservation (LaGeAC), at the São Paulo State University (UNESP), Jaboticabal city (São Paulo State, Brazil). Posteriorly, fish food with 50% of crude protein were gradually incorporated into the diet. In the fingerling stage, 1.2 mm pelleted feeds were used (40% of crude protein), being gradually replaced by 2 to 3 mm pelleted feeds (36% of crude protein) provided twice daily in 60 l tanks. Passive integrated transponder (PIT) tags were inserted into the intraperitoneum of individuals when they reached at least 5 g to maintain the pedigree information during the challenge experiments. Laterally, fish were kept in 800 l fiberglass tanks up to about 6 months post hatch.

### *Aeromonas hydrophila* challenge

The protocols for the *Aeromonas hydrophila* challenge, including the bacteria strain preparation, were similar to Mastrochirico-Filho et al. [6]. Briefly, a lethal dose in 50% of the individuals (LD50) was tested in 60 randomly selected fish from the same pacu families. Prior to the challenge, subsamples of populations were checked by microbiological tests to detect the presence of *A. hydrophila* and other pathogenic bacteria. In relation to the challenge test design, 381 juvenile individuals were distributed among three communal tanks (length = 2 m, width = 1 m, depth = 1 m), where approximately 10 individuals from each family (9.1, SD 1.3 individuals) were randomly distributed into each treatment tank (about 127 fish per tank), according to the sample size recommendation for disease resistance challenges proposed by Gjedrem and Baranski [48].

Before the bacteria inoculation, fish were placed for 1 min in a 3 l plastic washbasin containing benzocaine dissolved in water (10 mg/l), with continuous aeration, and weighed. The mean weight of the individuals prior to the bacteria inoculation time was 23.0 g (SD 9.06 g). When the individuals reached the dormant state, intraperitoneal inoculation of the predefined LD_50_ of *A. hydrophila* (8 × 10^5^ CFU/g body weight) were injected, according to protocols carried out by Mastrochirico-Filho et al. [6]. In parallel, 10 fish from each family (140 fish) were also used as control and kept in a separate but similar tank conditions (called as control tank). Individuals of the control tank were injected by intraperitoneal inoculation of phosphate-buffered-saline solution (PBS). An independent water recirculation system was maintained for both treatment and control tanks, similarly to Mastrochirico-Filho et al. [6].

Fish mortality events were registered all the time in the initial 3 days, and in 8-h intervals in the remaining days of the challenge experiment. Susceptible fish with clinical signs of *A. hydrophila* infection (e.g. disequilibrium, hemorrhage, isolation from the group) were recorded and removed immediately from the tanks. A subsample of dead fish was necropsied and suffered routine microbiological tests to confirm mortality by *A. hydrophila* and discard a possibility of infection caused by other pathogens.

At the end of the challenge period, surviving fish were checked externally for detection of clinical signs of the disease, and posteriorly euthanized using benzocaine.

### Genetic parameter estimation

Resistance was assessed as survival to the challenge test using the trait definitions of Mastrochirico et al. [6], such as Test survival (TS), which was analysed using a binary threshold (probit) model (THR) to account for the binary nature of the trait; and Time of death (TD), which was scored in hours (if fish survived to the end of challenge period, the time was recorded as 64.6 h) and it was analysed using a linear mixed model (LIN). The univariate animal models were defined as:

$$ {y}_{ij}=\mu +{t}_i+{w}_{ij}+{a}_{ij}+{e}_{ij}\ \left(\mathrm{LIN}\right) $$

where,*y*_*ij*_ was the phenotype for the fish *j*, in tank *i*; *μ* was the fixed effect of the overall mean; *t*_*i*_ was the fixed effect of the tank *i*; *w*_*ij*_ was the covariate of weight prior bacteria inoculation for fish *j*, in tank *i*; *a*_*ij*_ was the random animal genetic effect of individual *j,* in tank *i*; and *e*_*ij*_ was the random residual effect for the fish *j,* in tank *i*.

$$ \mathit{\Pr}\left({y}_{ij}\right)=\varPhi \left(\mu +{t}_i+{w}_{ij}+{a}_{ij}\right)\ \left(\mathrm{THR}\right) $$

where, *y*_*ij*_ was the phenotype (TS) for the fish *j,* in the tank *i*; *Φ*(·) was the cumulative standard normal distribution and the other parameters were similar to those described above.

THR and LIN models were fitted to estimate variance components by ASREML 3.0 package [49]. For all the models, the random animal genetic effect was assumed to be *N* (*0,* ${A\sigma}_a^2$), where *A* was the pedigree-based additive genetic kinship matrix among all the animals included in the population and ${\sigma}_a^2$ was the additive genetic variance. Random residuals for LIN were assumed to be *N* (*0,I* ${\sigma}_e^2$), where I was an identity matrix and ${\sigma}_e^2$ was the residual variance. For THR model, the residual variance on the underlying scale was set to 1. For both models, heritability was calculated as:

$$ {h}^2=\frac{\sigma_a^2}{\sigma_a^2+{\sigma}_e^2} $$

Where, ${\sigma}_a^2$ was the additive genetic variance and ${\sigma}_e^2$ was the residual variance.

### DNA extraction and construction of RAD libraries

For RAD-Seq analysis, fins from 391 individuals (373 offspring + 18 parents) were sampled for DNA extraction using the Dneasy Blood & Tissue QIAGEN kit. The quantification of extracted DNA (ng/μl) was measured by the Qubit fluorescence detector using the Qubit dsDNA BR Assay kit (Invitrogen, USA).

Each library was constructed with approximately 29 individuals identified with barcodes (14 libraries in total). Genomic DNA from each individual was digested by *Sbfl* enzyme. For each 11.35 μl of DNA (concentration at 10 ng/μl), 0.25 μl of *Sbfl* and 1.3 μl of NEBuffer 4 (New England Biolabs) were used. The enzyme digestion was performed at 37 °C for 1 h, followed by an inactivation period of 20 min at 80 °C.

Barcode adapters were ligated to the end of the DNA fragments following digestion with *Sbfl*, using the T4 DNA ligase (New England Biolabs). The ligation was incubated at 20 °C for 2 h, and then at 65 °C for 20 min. Following library construction, their quality was determined by PCR that combined 8.5 μl of water; 12.5 μl Phusion High-Fidelity Master Mix; 1 μl of RAD primer mix (forward and reverse) and 1 μl of digested DNA. The amplification was carried out with 18 cycles in a thermocycler (98 °C for 10 s, 65 °C for 30 s, 72 °C for 30 s) and 72 °C for 5 min. Size selection (300–500 bp) was carried out using excision of the appropriate band from gel electrophoresis. Purification was then carried out by the MinElute Gel Extraction kit, following the recommendations of the manufacturer. The concentration of each library was then normalized to 2.5 nm for sequencing by Edinburgh Genomics (University of Edinburgh, UK) on Illumina NovaSeq 6000 platform (flow cell Type S1 in 1 lane).

### Identification of SNPs from RAD-Seq technology

RAD sequences were filtered to discard those of low quality and posteriorly trimmed to 150 bp. Stacks 2.0 software [50] eliminated the ambiguous barcode sequences from the subsequent process. The remaining sequences were then separated according to the barcodes linked to the reads. Identical sequences based on similarity were filtered off using Dedupe Python Library [51] maintaining a single representative sequence. In relation of the main parameters that control locus formation by Stacks 2.0 software [50], the minimum depth of coverage (m) to create a putative allele (stack) was 3. The number of mismatches allowed between two alleles of sample (parameter M) was 3. Considering the RAD locus catalog containing parental individuals, the number of mismatches allowed between two alleles from the population to form a catalog was 3. Only loci present in at least 70% of individuals were considered to identify putative SNPs (call rate > 0.70). In order to differentiate putative SNPs from sequencing errors, Plink 1.9 software [52] was used to exclude SNPs with minor allele frequencies (MAF) lower than 0.05, *p*-value of Hardy-Weinberg disequilibrium (PHWE) lower than 1 × 10^− 6^. Additionally, SNPs with more than 20% of genotyping error rate (geno 0.2) and more than 10% of Mendelian error rate were also discarded. In relation to filter out individuals, samples presenting more than 30% of missing genotypes (mind 0.3) were excluded for linkage map construction.

### Linkage map and synteny analysis

Analysis of parentage assignment was performed by software Cervus3 [53, 54] using sex information of parents to confirm the pedigree. A linkage map was created using the modules of Lep-MAP 3 software [55]. *ParentCall2* confirmed reliable parental genotypes using joint information on offspring and parents. *Filtering2* was used to remove markers with significant segregation distortion (dataTolerance = 0.001). Markers were assigned into 27 linkage groups (LGs) (corresponding to the 27 pairs of chromosomes in pacu) by *SeparateChromosomes2*. In the LG assignment, an optimized LOD score of 8.6 was achieved binning the markers into LGs by adoption of LOD scores ranging from 4 to 15 [56]. The orphan markers were assigned to existing linkage groups (optimized LOD score = 6.7) using *JoinSingles2* to maximize the map-abilities of the total input of markers, and ordered the binned markers within linkage groups using *OrderMarkers2*. The generated linkage map was drawn using the *LinkageMapView* [57]. In order to verify the ordering of loci within the LGs belonging to the linkage map, correspondence synteny analysis between the chromosomes of blind cave tetra (*Astyanax mexicanus*) and the LGs for pacu was performed adopting the circos plots by Circa software (http://omgenomics.com/circa/).

### Genome-wide association study (GWAS)

GWAS for TS and TD traits were performed by genomic BLUP (GBLUP) and weighted genomic BLUP (wGBLUP) methods, adopting windows of 20 adjacent SNPs, and analyzed by BLUPF90 family of programs [58]. All available information on genotyped fish, including pedigree and phenotype records (*n* = 332 individuals) was considered. The animal models applied to the test was the same used for estimation of genetic parameters (above), but with addition of the genotype data. TD was analyzed as a linear trait using BLUPF90, whereas TS was analyzed as a threshold trait using THRGIBBS1F90. Gibbs sampling scheme was run considering one million iterations, of which the first 200,000 iterations were discarded. From the remaining 800,000 iterations one sample was saved from every 100 iterations.

The SNP based variance components and genomic estimated breeding values (GEBVs) were estimated, where *α* were a vector of random additive genetic effects with distribution ~*N* (*0,* ${G\sigma}_a^2$) and the numerator matrix *A* was replaced by a genomic relationship matrix *G* [59], that was constructed as:

$$ G= ZD{Z}^{\prime}\lambda $$

where, *Z* was the incidence matrix relating genotypes of SNPs with phenotypes (TS or TD), *D* was a diagonal inverse matrix with the expected variance for all SNPs, and *λ* was a weighting vector derived from the observed SNP frequencies. For GBLUP, matrix *D* equals identity matrix (*I*), while in wGBLUP, matrix *D* was estimated using the SNP effects using PREGSF90 and POSTGSF90. For this, GEBV ($\hat{a_g}$) were converted to SNP effects and the weights of SNP effect were refined being 1 for the first iteration, which means that all SNPs had the same weight. For the next iteration (2nd iteration), the weights were SNP-specific variances that were calculated through the estimates of the SNP allele-substitution effect of the preceding iteration and the corresponding SNP allele frequencies [60]. The equation for predicting SNP effects ($\hat{u}$) was [60, 61]:

$$ \hat{u}=\lambda DZ\acute{\mkern6mu}{G}^{-1}\hat{a_g} $$

where, $\hat{u}$ represented the vector of SNP effects and $\hat{a_g}$ the vector of GEBV of genotyped animals. With the results, the individual variance of SNP effects was estimated as [62]:

$$ {d}_{i\ \left(t+1\right)}={\hat{u}}_{j(i)}^22{p}_j\left(1-{p}_j\right) $$

where, ${\hat{u}}_{j(i)}^2$ was considered the square of the *j*th SNP marker effect in the *i*th individual, and *p*_*j*_ expressed the observed allele frequency for the second allele of the *j*th marker. After normalizing the matrix *D* and the weights of SNPs such that the total genetic variance remains constant, the percentage of additive genetic variance explained by each SNP window could be estimated as following:

$$ \frac{Var\left({u}_i\right)}{\sigma_a^2}\times 100\%=\frac{Var\ \left({\sum}_i^{20}{Z}_i\hat{a_{g_i}}\right)}{\sigma_a^2}\times 100\% $$

In total, 12,657 SNPs and 268 genotyped animals which passed on the quality control (call rate > 90%; MAF > 0.05) were analyzed. Manhattan plots based on the percentage of genetic variance explained by the *i*th SNP window was plotted by R-qqman [63] using R software.

SNP windows that explained more than 1% of the AGV for the traits were defined as suggestive QTL associated with *A. hydrophila* resistance [19, 64]. RAD-tag containing the highly associated SNPs were aligned against the *Pygocentrus nattereri* genome (a phylogenetically close fish species) using Nucleotide Basic Local Alignment Search Tool (BLASTn) to evaluate the presence of putative genes associated with the trait between the first and the last position of each 20 SNP window. The putative candidate genes identified were within or adjacent to each associated SNP.

## Data Availability

RAD sequencing raw reads supporting the conclusions of this manuscript have been deposited in the NCBI Sequence Read Archive (SRA) database “RAD-Seq of pacu challenged with *Aeromonas hydrophila*” (SRX8380423), under the accession number PRJNA634462 (https://www.ncbi.nlm.nih.gov/sra/?term=PRJNA634462). All scripts are available upon request to the corresponding author. Furthermore, the representative genome of blind cave tetra species (*Astyanax mexicanus*) used in genomic synteny analysis was obtained from the NCBI database (https://www.ncbi.nlm.nih.gov/genome/13073), under the accession number PRJNA237016.

## Abbreviations

*A. hydrophila*: *Aeromonas hydrophila*
AGV: Additive Genetic Variance
Am: *Astyanax mexicanus* chromosome
BLASTn: (nucleotide) Basic Local Alignment Search Tool
bp: Base pairs
CFU/g: Colony Forming Unit per gram
cM: Centimorgan
e.g.: *Exempli gratia*
g: Grams
GEBV: Genomic estimated breeding values
GWAS: Genome-Wide Association Study
h: Hours
Kb: Kilobases
l: Liters
LD50: Lethal dose in 50% of individuals
LG: Linkage Group
LIN: Linear model
m: Meter
m^3^: Cubic meter
MAF: Minor Allele Frequency
mg/kg: Milligram per kilogram
mg/l: Milligram per liter
min: Minutes
mm: Millimeters
ng/μl: Nanogram per microliter
nm: Nanometers
*P. mesopotamicus*: *Piaractus mesopotamicus*
QTLs: Quantitative Trait Loci
RAD-Seq: Restriction Site Associated DNA Sequencing
s: Seconds
SD: Standard Deviation
SNPs: Single Nucleotide Polymorphisms
GBLUP: Genomic Best Linear Unbiased Prediction
TD: Time of Death
THR: Threshold (probit) model
TS: Test Survival
w: Watt
wGBLUP: Weighted Genomic Best Linear Unbiased Prediction
μl: Microliter
